# Nipple aspirate fluid—A liquid biopsy for diagnosing breast health

**DOI:** 10.1002/prca.201700015

**Published:** 2017-06-26

**Authors:** Sadr‐ul Shaheed, Catherine Tait, Kyriacos Kyriacou, Joanne Mullarkey, Wayne Burrill, Laurence H. Patterson, Richard Linforth, Mohamed Salhab, Chris W. Sutton

**Affiliations:** ^1^ Institute of Cancer Therapeutics University of Bradford Bradford UK; ^2^ Bradford Teaching Hospitals NHS Trust Bradford UK; ^3^ The Cyprus School of Molecular Medicine The Cyprus Institute of Neurology and Genetics Nicosia Cyprus; ^4^ Ethical Tissue University of Bradford Bradford UK

**Keywords:** Breast cancer, Comparative proteomics, Liquid biopsy, Nipple aspirate fluid (NAF)

## Abstract

**Purpose:**

Nipple secretions are protein‐rich and a potential source of breast cancer biomarkers for breast cancer screening. Previous studies of specific proteins have shown limited correlation with clinicopathological features. Our aim, in this pilot study, was to investigate the intra‐ and interpatient protein composition of nipple secretions and the implications for their use as liquid biopsies.

**Experimental design:**

Matched pairs of nipple discharge/nipple aspirate fluid (NAF, *n* = 15) were characterized for physicochemical properties and SDS‐PAGE. Four pairs were selected for semiquantitative proteomic profiling and trypsin‐digested peptides analyzed using 2D‐LC Orbitrap Fusion MS. The resulting data were subject to bioinformatics analysis and statistical evaluation for functional significance.

**Results:**

A total of 1990 unique proteins were identified many of which are established cancer‐associated markers. Matched pairs shared the greatest similarity (average Pearson correlation coefficient of 0.94), but significant variations between individuals were observed.

**Conclusions and clinical relevance:**

This was the most complete proteomic study of nipple discharge/nipple aspirate fluid to date providing a valuable source for biomarker discovery. The high level of milk proteins in healthy volunteer samples compared to the cancer patients was associated with galactorrhoea. Using matched pairs increased confidence in patient‐specific protein levels but changes relating to cancer stage require investigation of a larger cohort.

**Abbreviations**
DCIS: ductal carcinoma in situ
ECM: extracellular matrix
EGFR: epidermal growth factor receptor
HV: healthy volunteer
IC: invasive carcinoma
NAF: nipple discharge/nipple aspirate fluid
PB: phyllodes tumor
PD: a patient with DCIS
PI: patient with invasive carcinoma
SCX: strong cation exchange chromatography

## 1 Introduction

Although the number of women, aged 40 or less, diagnosed with breast cancer is relatively low, they experience more aggressive forms of the disease with poorer clinical outcome 1, 2. They are often at high risk due to a genetic predisposition toward the disease, of which mutations in breast cancer type 1 susceptibility protein (*BRCA1)* and breast cancer type 2 susceptibility protein (*BRCA2)* tumor suppressive genes are the best characterized 3. Awareness of these variants, by germ line genetic testing, informs the patient of the life‐time risk of susceptibility to the disease compared to the general population, but it does not tell the patient when the disease will occur 4.

**Clinical Relevance**

There remains an unmet need to provide high risk premenopausal women with a regular and convenient alternative to mammography (reduced accuracy primarily due to breast density and jeopardizing patients with further exposure to radiation) for breast cancer screening. Tissue biopsies provide valuable diagnostic and prognostic information to support selection of treatments once tumors have been detected, and genomics has identified high penetrance genes to indicate those women at highest risk, but neither approach helps to detect the earliest manifestations of the disease. Detection of cancer biomarkers in blood receives much attention, but suffers from massive dilution in circulation compared to the diseased tissue of origin. Nipple discharge and nipple aspirate fluid are naturally occurring liquids secreted by the ducts and lobules, and hence have the potential to provide important diagnostic information regarding breast health. In this pilot study, we used proteomics to analyze paired NAF samples, to identify the protein profiles of volunteers and patients. The results indicate that matched pairs have similar protein composition but there are significant differences between individuals. The data can be diagnostic of breast health however a longitudinal patient study is required to establish protein changes that relate to cancer stage.

Mammography successfully detects breast cancer in postmenopausal women (98% sensitivity), but is less effective in younger women due to image obfuscation by breast density 5, 6. Also, mammography lacks the high specificity in differentiating between benign and malignant growths and also between microcalcifications associated with low grade ductal carcinoma in situ (DCIS), which may not require surgery, and higher grade DCIS that may progress to an invasive tumor 7, thereby resulting in overdiagnosis and overtreatment 8. Hence, the discovery of a mutation may result in elective surgery to remove both breasts or prophylactic administration of tamoxifen, with consequential side effects before the disease has occurred 9.

Therefore, new methods for the early detection of breast cancer are required to support high risk younger women. The search for diagnostic biomarkers of breast cancer has been extensive and proteomics strategies increasingly employed as part of the discovery process 10. Plasma is by far the most common biofluid used, but putative markers are massively diluted relative to the site of origin of the cancer, thereby reducing sensitivity 11. As an alternative, we have chosen to analyze secretions from the cells lining the ducts and lobules of the breast that manifest as a spontaneous nipple discharge or nipple aspirate fluid (herein collectively referred to as NAF), collected by massage or breast pump, thereby differentiating them from liquid biopsies obtained by lavage or needle extraction.

NAF comprises a diverse range of biological materials such as micronutrients (tocopherols, cholesterols, carotenes) 12, hormones (estradiol, estrone, progesterone, and testosterone) 13, carbohydrate antigens (Thomsen Friedenreich and Tn) 14, microRNA 15, and microbes 16 as well as proteins. It has multiple advantages as a liquid biopsy for detection of breast cancer: (i) premenopausal women are more likely to produce NAF than postmenopausal women where ductal atrophy may prevail 17, (ii) NAF expression is noninvasive, causing minimal discomfort compared to breast cancer screening procedures 18, (iii) it enables procurement of matched pairs of samples to provide an intraindividual comparison of the diseased with the healthy breast, (iv) biomarkers remain highly concentrated for analysis compared to blood and urine, and (v) minimal sample preparation is required, compared to tissues, thereby excluding yield‐reducing protein extraction steps. NAF collection can be challenging, often using microcapillaries, but recently Guthrie cards were employed, though subsequent proteomic analysis only identified high abundance proteins 19. NAF volumes are small, but protein concentrations are sufficient to enable analyses with state‐of‐the‐art mass spectrometric techniques. A number of strategies to determine the NAF proteome coverage have been undertaken many of which have been summarized by Pavlou et al., as part of a comparison with their own dataset of 854 proteins 20. More recently, similar studies by Brunoro et al. 21 and Kurono et al. 22 identified 557 and 372 proteins, respectively. Mostly these studies have focused on optimizing protein and peptide separation using single breast samples from each patient.

Our objective in this paper is to deal with the fundamental definitions of NAF composition in paired samples and determine if the proteins present constitute biologically and physiologically relevant information for diagnosing breast health.

## 2 Methods

### 2.1 Patients and sample collection

NAF samples were collected from breast cancer‐free (defined here as healthy) volunteers and breast cancer patients, who presented to Bradford Teaching Hospitals NHS Trust, in a prospective study between 2013 and 2016. All participants gave written informed consent to undergo bilateral nipple aspiration. The study protocol was approved by University of Bradford's Independent Scientific Advisory Committee (reference: application/13/051). Ethical approval was given by Leeds (East) Research Ethics Committee, reference 07/H1306/98+5. Before aspiration was attempted, the nipple was initially cleansed with an alcohol pad. NAF collection from cancer patients was performed under general anesthetic by the clinical team, prior to surgery, assisted by massaging the breast and the drop of liquid collected from the nipple surface using a sterile pipette. After collection, the samples were transferred to chilled, prelabeled tubes containing a freeze‐dried protease inhibitor cocktail mixture (Roche Diagnostics, Burgess Hill, UK), and frozen within 30 min of collection. NAF from healthy volunteers (HVs) was collected in a similar manner but by the individuals themselves. Where possible, NAF samples were collected from both breasts. From a bank of 112 NAF samples (comprising 55 pairs and 57 single samples), 15 pairs were selected for study and characterized for volume and color prior to further analysis (Supporting Information Table 1). Samples were centrifuged to remove particulate matter, the protein concentration measured using the Bradford assay and paired aliquots analyzed by SDS‐PAGE.

### 2.2 Proteomic analysis

An aliquot of each NAF sample was reduced, alkylated, and digested overnight using modified sequencing grade trypsin (see Supporting Information Materials and Methods). Digests were desalted, lyophilized, and then resuspended in 10 mM KH_2_PO_4_ in 25% v/v acetonitrile, 0.01% w/v sodium azide, pH 3.0. The digests were subject to strong cation exchange chromatography (SCX) with peptides fractionated using stepwise increases in potassium chloride concentration. The SCX desalted fractions were desalted and lyophilized.

### 2.3 Fusion orbitrap analysis

The lyophilized SCX fractions were resuspended in 0.1% FA and analyzed in triplicate on a nano‐LC UltiMate 3000 capillary HPLC system coupled to an Orbitrap Fusion™ Tribrid™ Mass Spectrometer (see Supporting Information Materials and Methods). Samples were applied to a C_18_, 300 μm × 5 mm, 5 μm diameter, 100 Å PepMap precolumn before transfer to a C_18_, 75 μm × 50 cm, 2 μm diameter, 100 Å PepMap column. A binary solvent system was used for chromatographic separations composed of 0.1% FA in 2% acetonitrile and 0.1% FA in 100% acetonitrile. Data‐dependent acquisition using dynamic scan management was performed, generating full MS spectra in the Orbitrap and MS/MS acquisition in the ion‐trap.

### 2.4 Data analysis

MS/MS fragment mass lists were searched using Proteome Discoverer version 2.1 and Mascot software version 2.4 (see Supporting Information Materials and Methods). Only Master Proteins (i.e., containing at least one unique peptide) were accepted. Protein quantitation was defined as the sum of the peak areas of the three strongest parent signals. Quantitation was normalized for cross‐sample comparison. Pearson correlation coefficient calculated to determine gross similarities of paired samples, Student *t*‐tests used to identify significantly (*p* < 0.05) expressed proteins and FunRich 2.1.2 used to compare proteomes and identify common proteins. Database for Annotation, Visualization and Integrated Discovery (DAVID) version 6.8 was used for functional annotation 23, STRING version 10.0 for protein–protein interaction analysis 24, TMHMM Server v. 2.0 for membrane association, the Plasma Proteome Database used for comparison with the NAF proteome, the Kyoto Encyclopedia of Genes and Genomes (KEGG) database to identify metabolic pathways, and the Early Detection Research Network (EDRN) to identify breast cancer biomarkers.

## 3 Results

### 3.1 NAF sample characterization

**Figure 1:**
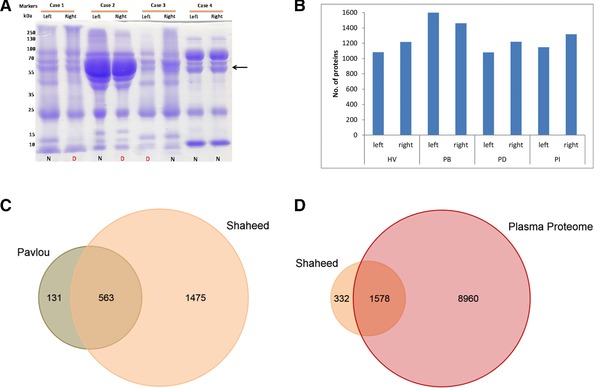
(A) SDS‐PAGE analysis of matched pairs of NAF samples from three IC patients (cases 1–3) and one HV (case 4). N, NAF samples from normal breast; D, NAF sample from diseased (benign, DCIS or IC) breast. Arrow indicates the position of serum albumin. (B) Number of identified protein (≥2 PSMs) by 2DLC MS analysis of eight NAF samples (four matched pairs, HV, PB, PD, PI). Venn diagrams illustrating (C). overlapping identities with the data from the NAF study by Pavlou et al 20, and D. overlapping identities with the Human Plasma Proteome database.

Our preliminary objective was to characterize matched pairs of NAF using basic biochemical procedures, measuring the protein concentration and amount, and visualization by SDS‐PAGE, which has not been reported previously. NAF samples from 100 breast cancer patients and HVs were grouped into four clinical stages—invasive carcinoma (IC), DCIS, benign lesions, and healthy. From these, 15 pairs (two noncancer, two benign, one DCIS, and 10 ICs) were selected and characterized for volume (varying from 4 to 500 μL) and protein concentration (3–70 mg/mL (Supporting Information Table 1). Samples were analyzed by SDS‐PAGE demonstrating that, in the majority of cases, pairs from the same individual had similar profiles (Fig. 1A, Supporting Information Fig. 1 A–C). Some cases exhibited a dominant serum albumin band, suggesting a high plasma content (Fig. 1A, Case 2), while others had a relatively low albumin presence (Fig. 1A, Cases 1, 3, and 4). Based on sample color there was no indication of blood in the former group, no apparent correlation with disease compared to healthy, and hence was not indicative of tissue damage or tumor invasiveness. Four matched pairs, a HV (Case 4, HV), a patient with benign phyllodes tumor (Case 9, PB), a patient with DCIS (Case 10, PD) and a patient with IC (Case 12, PI) (Supporting Information Table 1), were selected for proteomic analysis, based on similar protein concentration and protein quantity (to minimize samples preparation variation)

### 3.2 NAF proteomic analysis

For NAF to be a useful clinical sample it was important to establish the quality and complexity of proteomic data that can be achieved using 2D‐LC/MS separation. From 2D‐LC/MS analysis of all eight samples, a total of 1990 gene products were identified (*p* < 0.05) (Supporting Information Table 2), with an average of 1265 proteins per sample (SD ± 185) (Fig. 1B). Prior to this study, the most complete proteomics profile of NAF was that of Pavlou et al. 20. Comparison with our dataset, based on gene identity (691 entries Pavlou et al., and 1919 for our set, excluding immunoglobulin isoforms), indicated an overlap of 563 proteins (Fig. 1C), however our current study illustrated substantial progress in NAF characterization identifying 1374 new proteins not previously seen in NAF.

As plasma is by far the most commonly used and most completely characterized liquid biopsy for diagnosing disease, including breast cancer 10, we wanted to establish if the NAF proteome is likely to provide unique insights. The Plasma Proteome Database comprises the collated quantitative data for 10 546 proteins that have been detected in plasma and serum using immunoassays or mass spectrometric techniques (http://www.plasmaproteomedatabase.org/) 25. A comparison of the NAF profile with the plasma proteome identified 1578 proteins in common (Fig. 1D), however 332 proteins (21% of the total NAF profile) were unique to NAF indicating excellent potential to provide molecular information specific to breast health.

**Figure 2:**
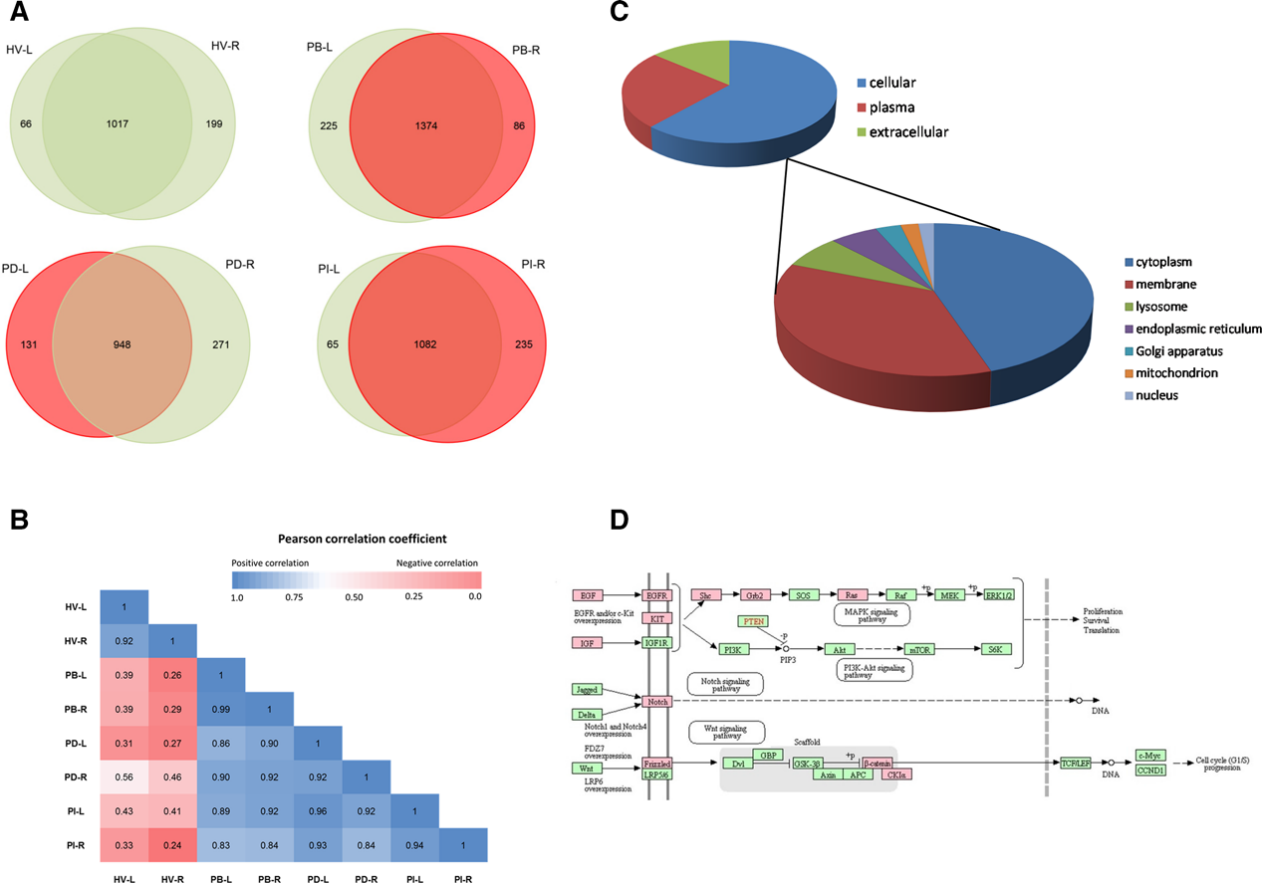
(A) Comparison of the matched NAF protein profiles. (B) Pearson correlation coefficient for each NAF profile compared to each other, based on median normalized log_2_ peak area for each protein. (C) Normal cellular localization of the 567 proteins common to all the NAF samples. (D) NAF proteins detected in breast cancer signaling pathways, highlighted in red with KEGG user data mapping function (http://www.genome.jp/kegg/)

Pairwise comparison of the NAF proteome complements exhibited more than 50% likeness in composition (Supporting Information Fig. 2). Profiles for matched pairs from the same individual showed the greatest similarity with 1017 of a total of 1282 proteins, 1374/1685, 948/1350, and 1082/1382 common for HV, PB, PD, and PI pairs, respectively (Fig. 2A). When the quantitative data (sum of the peak areas of the 3 most abundant unique peptides per protein) were included, the bilateral pairs, again showed greatest positive correlation (Pearson correlation coefficient values of 0.92 to 0.99) (Fig. 2B). The sample pair for the HV, however, showed the lowest correlation with the other three cases, suggesting a unique constitution.

### 3.3 NAF composition

The 567 proteins common to all eight 2D‐LC analyses were manually categorized based on location using UniProt (release 1026_07) into (i) intracellular, (ii) extracellular stroma surrounding the breast cells, or (iii) plasma (Fig. 2C). Intracellular proteins were further subdivided into cytoplasmic, membrane‐linked (i.e., containing transmembrane domain, GPI or lipid anchors), or organelle‐specific location. Of the common proteins, 25% are normally found in plasma, 14% function within the extracellular space, and 61% are from cell components. Of the 346 cellular proteins, 45% are normally found in the cytoplasm, 36% are membrane associated, 7% lysosome, 8% endoplasmic reticulum/Golgi apparatus, 2% mitochondrion, and 2% in the nucleus. The nuclear and mitochondrial proteins were particularly underrepresented, normally contributing 14 and 6% of the total human proteome, respectively 26, 27.

Submission of the total NAF proteome to the TMHMM transmembrane protein search engine identified 415 proteins with transmembrane regions and a further 147 proteins with GPI‐anchor, N‐terminal, or cys‐modified lipid attachments were identified from UniProt. Hence, 27% of the proteins present in NAF are normally found anchored in membranes and consequently provide a valuable source of information regarding cell integrity within breast tissue. Using STRING analysis, 74 of the membrane proteins were linked to cell adhesion (FDR 2.88 × 10^−24^), 50 proteins involved in glycoprotein metabolism (FDR 2.88 × 10^−24^), and 86 proteins have receptor activity (FDR 1.04 × 10^−21^).

Although some are case specific, among the receptors detected were those associated with cancer signaling pathways, including epidermal growth factor receptor (EGFR), transforming growth factor beta receptor type II and III, hepatocyte growth factor receptor (HGFR), vascular endothelial growth factor receptor (VEGFR1), mast/stem cell growth factor receptor (KIT), and receptor tyrosine‐protein kinase Erbb‐3 (Fig. 2D). Furthermore, 21 mitogenic growth factors were detected including pleiotrophin, macrophage colony‐stimulating factor 1 (CSF1), transforming growth factor beta, and those produced by cancer‐associated fibroblasts; proepidermal growth factor (EGF), insulin‐like growth factors I (IGF1) and II, and platelet‐derived growth factors C (PDGF‐C) and D (Table 1). Downstream processes activated by ligand‐receptor binding, including protein kinases and protein phosphatases that play a role in MAPK‐, NOTCH‐, and Wnt‐signaling pathways, were also identified.

**Table 1:** Selected mitogenic factors and receptors detected in NAF samples

Gene ID	Description	Accession	Score Mascot	Coverage	No. of spectra	No. of unique peptides	MW (kDa)	Calc. p*I*	Frequency	Comments	Detected in plasma
**Receptors**											
CD22	B‐cell receptor CD22	P20273	87	1.4	45	1	95.3	6.7	6	All except HV	MS
NTRK2	BDNF/NT‐3 growth factors receptor	Q16620	988	3.9	54	3	91.9	6.5	8		MS
MET	Hepatocyte growth factor receptor	P08581	54	2.5	10	2	155.4	7.3	2		ELISA
IFNAR1	Interferon alpha/beta receptor 1	P17181	305	2.3	17	1	63.5	5.8	5		MS
IL1R1	Interleukin‐1 receptor type 1	P14778	405	9.0	53	3	65.4	7.8	6	All except HV	MS
IL13RA1	Interleukin‐13 receptor subunit alpha‐1	P78552	278	5.2	18	2	48.7	6.0	4		MS
IL6ST	Interleukin‐6 receptor subunit beta	P40189	4320	12.0	307	8	103.5	6.0	8		ELISA
IL7R	Interleukin‐7 receptor subunit alpha	P16871	260	7.0	23	3	51.5	5.4	2	HV only	MS
LIFR	Leukemia inhibitory factor receptor	P42702	1751	13.6	176	12	123.7	5.7	6	all except HV	IA
LDLR	Low‐density lipoprotein receptor	P01130	331	6.5	23	3	95.3	5.1	6		MS
KIT	Mast/stem cell growth factor receptor Kit	P10721	301	4.6	39	4	109.8	7.0	4	HV and PI pairs	ELISA
PGRMC1	Membrane‐associated progesterone receptor component 1	O00264	67	7.7	8	2	21.7	4.7	3		MS
NRP1	Neuropilin‐1	O14786	2910	23.7	240	15	103.1	5.9	7		MS ‐ MRM
ERBB3	Receptor tyrosine‐protein kinase erbB‐3	P21860	164	1.8	15	1	148.0	6.6	5		MS
TGFBR2	TGF‐beta receptor type‐2	P37173	49	2.6	24	1	64.5	5.9	7		MS
TGFBR3	Transforming growth factor beta receptor type 3	Q03167	279	9.4	26	5	93.4	5.7	2		MS
**Mitogenic factors**											
IGF1	Insulin‐like growth factor I	P05019	35	5.1	4	1	21.8	9.7	2	IC pair only	ELISA
IGF2	Insulin‐like growth factor II	P01344	31	5.0	3	1	20.1	9.3	2		ELISA
IL8	Interleukin‐8	P10145	123	16.2	13	1	11.1	8.8	2	PD only	CIA
IL19	Interleukin‐19	Q9UHD0	155	22.0	39	3	20.4	7.7	2	HV only	MS
IL34	Interleukin‐34	Q6ZMJ4	358	6.6	26	1	27.5	7.2	8		Not detected
MYDGF	Myeloid‐derived growth factor	Q969H8	177	15.6	14	2	18.8	6.7	4		MS
PGF	Placenta growth factor	P49763	82	19.0	30	2	24.8	8.2	6	All except HV	ELISA
PDGFC	Platelet‐derived growth factor C	Q9NRA1	1960	15.7	117	5	39.0	6.1	8		Not detected
PDGFD	Platelet‐derived growth factor D	Q9GZP0	968	10.8	51	3	42.8	8.0	8		Not detected
PTN	Pleiotrophin	P21246	1536	23.8	92	3	18.9	9.6	8		MS
EGF	Pro‐epidermal growth factor	P01133	17275	28.4	1201	25	133.9	5.9	8		MIA
TGFB1	Transforming growth factor beta‐1	P01137	41	3.3	2	1	44.3	8.5	2		ELISA
TGFB2	Transforming growth factor beta‐2	P61812	71	12.8	12	3	47.7	8.5	4		Not detected
TGFB3	Transforming growth factor beta‐3	P10600	39	3.6	2	1	47.3	8.0	1		MS‐MRM

In addition, cell adhesion proteins were common in NAF including CEACAMs 1, 5, 6, and 8, NCAM2, BCAM, ALCAM, ECAM, MCAM, and ICAM1, 14 cadherin/protocadherin proteins, and five integrin subunits. Basement membrane proteins, extracellular matrix (ECM) proteins, and proteoglycans, including laminins, mucins, collagens, and fibulins, which play an important role in cell‐ECM and cell–cell interactions, were prevalent (see Supporting Information Table 2). Laminins form a complex comprising a heterotrimer of alpha, beta, and gamma subunits linked by disulphide bridges, constitute a key component of basement membranes and have an essential role in the structure and function of ECM 28. Of the five laminin isoforms detected, alpha5, beta2, and gamma1 were the most abundant in all NAF samples, indicating the expression of the specific heterotrimer laminin‐11 (or laminin‐521) normally found in the glomerular basement membrane of the kidney, in the neuromuscular synaptic cleft and in placenta 29. Overall NAF samples comprised of a high proportion of proteins functioning in the tumor microenvironment, which are responsible for cancer cell behavior including proliferation, survival, adhesion, migration, and invasion. Cancer‐associated fibroblast markers (neprolysin, matrix metalloproteinase 9 [MMP9], tenascin‐C), markers of epithelial‐mesenchymal transition, a prerequisite to metastasis (TGFβ1, cadherin 1/E‐cadherin, fibronectin, vimentin, cytokeratin 8, and cytokeratin 18) and breast cancer stem cell markers (CD44, CD133) were also detected in all NAF samples.

Mannello et al. identified the importance of exploring NAF for established biomarkers such as urokinase‐dependent plasminogen activator (uPA) and plasminogen activator inhibitor (PAI‐I), particularly for their role in ECM turnover associated with cancer invasiveness 30. Our analysis of NAF identified approximately 100 proteolytic enzymes, including uPA, MMP9, and matrilysin, which function to modulate stromal composition. Within this group were ten members of the kallikrein family, including prostate specific antigen (PSA/KLK3) (see Supporting Information Table 2). Previously, an inverse correlation of PSA levels (measured by immunofluorometric assay) in NAF, with progressive breast cancer (DCIS to metastatic), has been described 31, 32.

There were six proteins (kallikrein 6, ATP‐binding cassette sub‐family C member 11, secretoglobin family 3A member 1, mammaglobin‐A, prolactin‐inducible protein [PIP], and mucin‐like protein 1) that are most strongly expressed in breast tissues (compared to all other tissues), and although not cancer specific, may prove useful indicators of breast health. PIP has previously been explored as a NAF‐derived biomarker of breast cancer by proteomics and ELISA methods, and expression found to correlate with pre‐/postmenopausal status and cancer stage 33.

The National Cancer Institute has coordinated the research of many institutions to accelerate the identification and validation of early stage cancer testing and detection (https://edrn.nci.nih.gov/). Of the 195 breast cancer proteins and genes under investigation by the NCI Early Detection Research Network, 46 are present in NAF of which 22 were detected in all eight samples and seven were not found in plasma (Table 2). Among the candidate biomarkers detected in NAF were C‐C motif chemokine 28, CSF1, EGFR, VEGFR1, VEGFA, ICAM1, KIT, HGFR, MMP9, metalloproteinase inhibitor 1 (TIMP1), osteopontin, and Toll‐like receptor 2.

**Table 2:** Biomarkers under investigation by the NCI Early Detection Research Network

Gene ID	Description	Accession	Mascot Score	No. of spectra	No. of unique peptides	Plasma	Case profile
ADH5	Alcohol dehydrogenase class‐3	P11766	252	25	4	MS	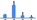
AKR1C2	Aldo‐keto reductase family 1 member C2	P52895	612	51	4	ND	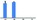
AKR1B1	Aldose reductase	P15121	184	42	1	MS	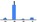
ALPL	Alkaline phosphatase, tissue‐nonspecific isozyme	P05186	3082	168	7	MS	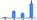
ANXA1	Annexin A1	P04083	9026	362	16	MS	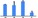
CDH1	Cadherin‐1	P12830	10468	590	14	MS	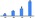
CEACAM5	Carcinoembryonic antigen‐related cell adhesion molecule 5	P06731	1328	104	2	MS	NQ
CCL28	C‐C motif chemokine 28	Q9NRJ3	318	30	1	ND	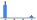
MYCBP	C‐Myc‐binding protein	Q99417	25	9	2	MS	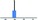
CRP	C‐reactive protein	P02741	38	8	1	MS/ELISA	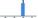
ENG	Endoglin	P17813	2738	196	9	MS/ELISA	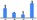
EFNA5	Ephrin‐A5	P52803	660	121	6	ND	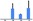
EGFR	Epidermal growth factor receptor	P00533	132	18	3	MS/IA	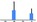
STOM	Erythrocyte band 7 integral membrane protein	P27105	22404	1392	14	MS	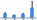
FABP5	Fatty acid‐binding protein, epidermal	Q01469	554	60	4	MS	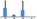
GPI	Glucose‐6‐phosphate isomerase	P06744	6832	334	11	MS	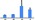
GSTM1	Glutathione S‐transferase Mu 1	P09488	7599	544	7	MS	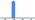
GSTM2	Glutathione S‐transferase Mu 2	P28161	2427	197	6	ND	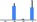
GNB4	Guanine nucleotide‐binding protein subunit beta‐4	Q9HAV0	317	77	2	ND	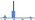
MET	Hepatocyte growth factor receptor	P08581	54	10	2	MS	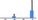
ITGB1	Integrin beta‐1	P05556	32	7	2	MS	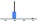
ICAM1	Intercellular adhesion molecule 1	P05362	5942	337	12	MS/ELISA	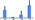
GLO1	Lactoylglutathione lyase	Q04760	253	24	3	MS	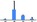
LBP	Lipopolysaccharide‐binding protein	P18428	15772	899	9	MS	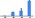
CSF1	Macrophage colony‐stimulating factor 1	P09603	17423	1181	7	MS	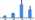
KIT	Mast/stem cell growth factor receptor Kit	P10721	301	39	4	MS	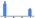
MMP9	Matrix metalloproteinase‐9	P14780	503	32	5	MS	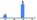
TIMP1	Metalloproteinase inhibitor 1	P01033	4627	378	7	MS	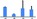
NDUFA10	NADH dehydrogenase [ubiquinone] 1 alpha subcomplex subunit 10, mitochondrial	O95299	107	7	1	MS	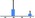
PVRL4	Nectin‐4	Q96NY8	360	54	4	ND	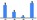
SPP1	Osteopontin	P10451	11049	982	13	MS/ELISA	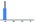
PRDX4	Peroxiredoxin‐4	Q13162	3107	334	9	MS	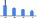
PDCD6IP	Programmed cell death 6 interacting protein	Q8WUM4	20305	1354	33	MS	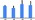
S100A4	Protein S100‐A4	P26447	33	17	1	MS	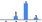
RAC1	Ras‐related C3 botulinum toxin substrate 1	P63000	1097	177	5	MS	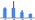
RAB13	Ras‐related protein Rab‐13	P51153	961	75	2	MS	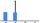
RAB5A	Ras‐related protein Rab‐5A	P20339	1219	131	3	MS	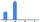
SERPINB3	Serpin B3	P29508	173	18	3	MS	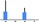
SSBP1	Single‐stranded DNA‐binding protein, mitochondrial	Q04837	33	5	2	MS	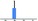
TLR2	Toll‐like receptor 2	O60603	2408	199	15	MS	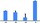
RHOA	Transforming protein RhoA	P61586	3306	364	2	ND	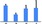
TNFRSF11B	Tumor necrosis factor receptor superfamily member 11B	O00300	27	2	1	MS/ELISA	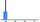
TNFRSF1A	Tumor necrosis factor receptor superfamily member 1A	P19438	107	5	1	MS/ELISA	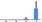
VEGFA	Vascular endothelial growth factor A	P15692	69	32	1	ELISA/IA	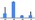
FLT1	Vascular endothelial growth factor receptor 1	P17948	295	58	1	ELISA	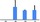
ATP6AP1	V‐type proton ATPase subunit S1	Q15904	3922	202	6	MS	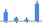

**Figure 3:**
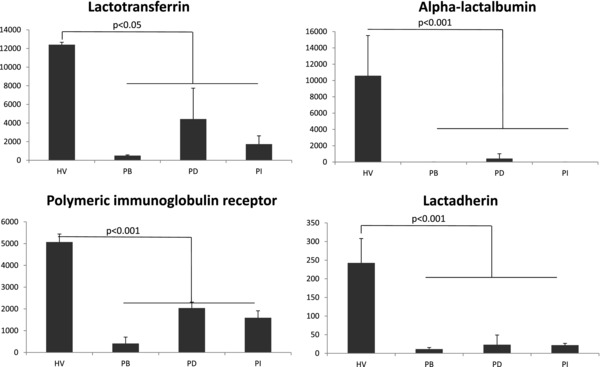
Representative profiles of milk proteins significantly increased in the HV compared to the three cancer patients (PB, PD, and PI). Quantitation is defined as the ratio of area for the specific protein relative to the median of the area for the protein complement. Each value is the average of the values for the left and right breast measurements.

ND, not detected; NQ, not quantified; case profile description, see Fig. 3.

Cytochrome P450 3A4 (CYP3A4) was detected at low abundance in two NAF samples, right breast of the benign patient and left breast of the IC patient, and verified by western blotting (Supporting Information Fig. 3). CYP3A4 plays an important role in converting tamoxifen to N‐desmethyl‐4‐hydroxytamoxifen, which has a 30‐ to 100‐fold higher affinity for estrogen receptor than tamoxifen 34. CYP3A4, measured by immnuohistochemistry in normal and cancer breast tissue biopsies was found to be prognostic for patient response to docetaxel 35, 36 and by activity assay and western blot to correlate with ifosfamide activation 37. The presence of CYP3A4 in NAF provides a unique opportunity to screen for patients who are most likely to respond to prophylactic tamoxifen treatment.

### 3.4 Diagnostic application

One of our objectives was to understand whether analyzing matched pairs would provide a more specific approach to detecting disease compared to normal. SDS‐PAGE showed that most matched pairs have similar protein band patterns, which was corroborated by the high correlation of proteomics profiles. While some proteins were significantly different in bilateral samples, a more extensive longitudinal study is required to determine statistically valid differences between disease and healthy breast of an individual.

Nevertheless, the proteomic profiling of the HV (Case 1) provided important diagnostic feedback relating to the cause of nipple discharge. As already noted, the proteomic profiles from Case 1 exhibited least correlation with the three disease cases (Fig. 2B). A two‐tailed Student *t*‐test of HV, using the average normalized sum of the three strongest peak areas for each protein, from two breast analyses, compared to the equivalent data for the three cancer samples, identified 331 proteins that were present at significantly different levels (*p* < 0.05)(Supporting Information Table 3). The proteomic signature for Case 1 indicated high levels of milk proteins. Of the 20 most abundant proteins observed by Beck et al., the most complete proteome study of human milk to date, 17 were also present in the top 50 most abundant detected in the NAF samples of Case 1 38 (Fig. 3). Case 1 presented at the outpatient clinic with a spontaneous milky pus discharge when either nipple was squeezed. The reproductive history of the volunteer, aged 48, indicated, she had achieved parity three times (with the first birth at age 38). Small amounts of milk or serous fluid expression can persist for months or years after weaning, but Case 1 did not engage in breastfeeding. Further investigation of her medical records, however, indicated that she had been prescribed amitriptyline, for depression and stress management, and omeprazole and lansoprazole for gastric esophageal reflux. In rare cases, these may cause breast tissue enlargement and nipple discharge, which is associated with galactorrhea rather than cancer 39.

## 4 Conclusions

In this study, NAF samples were collected by manual massage techniques prior to surgery or when the patients presented with a natural discharge at outclinic. Of those volunteers who consented, the success rate in NAF expression was approximately 50%. In order to provide a suitable screen approach for breast cancer specific biomarkers, increased success in expression and collection will be required. Improved expression rates can be achieved with the aid of oxytocin nasal spray 18, 40 as well as application of manual or mechanical pumps that are normally used for milk expression by mothers with preterm infants 41.

Our aim was to determine whether NAF has the potential to provide diagnostic value in screening for breast cancer. The possibility of using an internal control sample from the healthy breast for comparison with the diseased breast was considered. The complement of proteins in matched pairs showed strong similarity, probably due to transport through cross‐lymphatic drainage, which may make symptomatic differentiation challenging. Conversely, single samples from patients, where expression is poor, would be sufficient for clinical diagnosis of disease‐related biomarkers. In this respect, we have identified double the number of proteins previously detected in NAF, including 300 not found in plasma and 24% of the markers currently part of the NCI Early Detection Research Network studying breast cancer. The current breast cancer markers, growth factors, and receptors which have been detected in plasma, required a number of independent approaches (immunoassays and MS), whereas we have the potential to develop a single quantitative, multiplexed, target method by multiple reaction monitoring MS, utilizing valuable NAF samples efficiently. Furthermore, the composition of NAF was dominated by proteins representative of the basement membrane, extracellular milieu, and interstitial fluid surrounding breast cells, with roles in tissue stability, cell adhesion, and cell–cell communication. Future NAF proteomic analysis will aim to investigate if changes in the proteins correlates with stromal disruption and degradation as cancer cells proliferate and migrate into the surrounding normal tissue environment. Overall, the study has identified many physiologically and oncologically important proteins that warrant a more expansive study of a larger cohort of patients and HVs.

The authors have declared no commercial conflicts of interest.

## Supporting information

Materials and Methods

Supplementary Figures 1, A – C. SDS PAGE analysis of matched pairs of NAF samples from additional patients and a healthy volunteer (Cases 5 to 15), N = NAF samples from normal breast, D = sample from diseased breast. Arrow indicates the position of serum albumin
Supplementary Figure 2. Correlation of NAF profile pairs, HV = healthy volunteer, PB = patient with benign lesion, PD = patient with DCIS, PI = patient with invasive carcinoma
Supplementary Figure 3. Western blot analysis of β‐actin and CYP3A4 presence in the NAF samples. HV = healthy volunteer, PB = patient with benign lesion, PD = patient with DCIS, PI = patient with invasive carcinoma. N = healthy/normal breast, T = tumour

Supplementary Table S1

Supplementary Table S2

Supplementary Table S3
