# Supplementary material for: Nipple aspirate fluid—A liquid biopsy for diagnosing breast health
Source: Proteomics Clin Appl. 2017 Jun 26;11(9-10):1700015. doi: 10.1002/prca.201700015 (PMC5638085; doi:10.1002/prca.201700015)
Supplement: Supplementary file 1 — Materials and Methods [file PRCA-11-na-s001.docx]

**Materials and Methods**

Unless otherwise stated, reagents were purchased from Sigma-Aldrich (Gillingham, UK).

**Patients and sample collection**

NAF samples were collected from healthy* volunteers and breast cancer patients

*The clinicians determined that the patients were considered breast cancer-free (and therefore healthy) if they were completely asymptomatic or didn’t have, at presentation, red flag signs on examination (such as malignant feeling mass, bloody nipple discharge, skin changes, breast shape change). Those patients who had any symptoms were assessed further using triple assessment method routinely practiced at breast clinics in the UK (clinical examination + imaging +/- pathology if needed) and if negative, were deemed healthy.

**NAF Sample preparation**

The samples were centrifuged for 1 minute to remove particulate matter and the supernatants collected. The protein concentration was measured using the Bradford assay (Bio-Rad Laboratories, Hemel Hempstead, UK) and paired samples (20μg) analysed by SDS-PAGE as described previously [1]. Gels were stained with PhastGel Blue R (in 5% acetic acid, 50% methanol) for protein detection.

**Proteomic analysis**

An aliquot of each NAF sample (200 μg) was reduced with 50 mM dithiothreitol for 15 minutes at 60°C, alkylated with 100mM iodoacetamide at ambient temperature, in the dark, for 15 minutes, and digested by modified sequencing grade trypsin (Fisher Scientific, Loughborough, UK); protease-to-protein mass ratio of 1:20 (w/w) at 37°C for 20 hrs. After digestion, each sample was desalted on an Isolute C_18_ RP LC column (Kinesis Ltd, St Neots, UK) and lyophilized.

**SCX Chromatography Peptide fractionation**

Trypsin-digested NAF samples were re-suspended in Strong Cation Exchange Chromatography (SCX) loading buffer (10mM KH_2_PO_4_ in 25% v/v acetonitrile [ACN], 0.01% w/v sodium azide, adjusted to pH3.0) and added to an Isolute SCX column (Kinesis), equilibrated with SCX loading buffer. Peptides were eluted in 12 fractions with stepwise increasing potassium chloride concentration from 0 to 1000mM (0, 30, 60, 90, 120, 150, 200, 250, 300, 350, 500, 1000mM). Eluted fractions were diluted with 2% v/v ACN, 0.05% v/v formic acid (FA), desalted on an Isolute C_18_ RP LC column (Kinesis) and lyophilised.

**Fusion Orbitrap analysis**

The lyophilised SCX fractions were re-suspended in 10μl of 0.1% FA and analysed in triplicate (3μl/injection) on a nano-LC UltiMate 3000 capillary HPLC system coupled to an Orbitrap Fusion™ Tribrid™ Mass Spectrometer (ThermoFisher Scientific, Hemel Hempstead, UK). Samples were applied and washed for 4 minutes on a C_18_, 300 μm × 5 mm, 5 μm diameter, 100 Å PepMap pre-column at 25μl/min (ThermoFisher) before transfer to a C_18_, 75 μm × 50 cm, 2 μm diameter, 100 Å PepMap column run at a flow rate of 300nl/min (ThermoFisher). A binary solvent system was used for chromatographic separations with solvent A composed of 0.1% FA in 2% acetonitrile and solvent B was 0.1% FA in 100% acetonitrile. Peptides were eluted with a linear gradient to 5−30% mobile phase B over 70 minutes then 30-45% mobile phase B for 15 minutes. The column was washed for 15 minutes with 80% solvent B, before re-equilibration to 5% B. Orbitrap Fusion parameters were as follows: for full MS spectra, the scan range was m/z 350–1500 with a resolution of 120,000 at m/z 200. All MS/MS acquisition was performed on Ion-trap, in top speed mode with 3s cycle time, dynamic exclusion (±5 ppm) of 60 seconds and intensity threshold 5000. Ions with charge states 2+ to 7+ were sequentially fragmented by collision-induced dissociation (CID) with a normalized collision energy of 35%. A maximum of 200 ms ion injection time was allowed.

**Data Analysis**

MS/MS fragment mass lists were searched, via Proteome Discoverer version 2.1 (ThermoFisher) using Mascot software version 2.4 (Matrix Science, London, UK) with a percolator (strict FDR of 0.01 and a relaxed FDR of 0.05) against SwissProt version 2016 containing 552,259 human protein sequences with search parameters: trypsin digestion, 2 missed cleavages, variable modification of methionine oxidation, fixed modifications of cysteine (carbamidomethylation), precursor mass tolerance of 10 ppm, MS/MS fragmentation mass tolerance of 0.5 Da and a 95% confidence interval threshold (p < 0.05, Mascot score ≥23). Non-redundant protein profiles for each NAF sample were created by combining the corresponding LC−Fusion datasets. The list of protein identifications were assessed manually, and only Master Proteins (i.e. contain unique peptides) were accepted.

Protein quantitation was defined as the sum of the peak areas of the three strongest parent signals. To allow comparison of sample protein profiles, quantitation of each protein was normalised relative to the median peak area sum of the whole protein complement in the sample. Pearson correlation coefficient was calculated using PRISM 6.0 software (GraphPad Software), to determine gross similarities of paired samples. To identify cases-specific (healthy vs disease [HV vs PB,PD,PI], protein expression, averaged data of paired (left and right breast) samples was used. Student t-tests were undertaken using Excel 2010 to identify significantly (p<0.05) expressed proteins. A functional Enrichment analysis tool, FunRich 2.1.2 (http://www.funrich.org/) was used to compare proteomes of NAF samples and identify a group of common proteins. Proteins which met the required thresholds for identification were submitted to Database for Annotation, Visualization and Integrated Discovery (DAVID, version 6.8) analysis [2]. Default settings were used for functional annotation with p-values of <0.05, deemed significant. Protein-protein interaction analysis was performed using STRING version 10.0 (http://string-db.org/)[3]. Proteins were also subject to analysis in TMHMM Server v. 2.0 (http://www.cbs.dtu.dk/services/TMHMM/) [4], to determine those which were normally located in membranes. The Plasma Proteome Database (http://www.plasmaproteomedatabase.org/) was used for comparison of the NAF with the plasma proteome, the Kyoto Encyclopaedia of Genes and Genomes (KEGG) pathway database (http://www.genome.jp/kegg/pathway.html) used to identify those pathways associated with the NAF profiles, and the Early Detection Research Network (EDRN) (https://edrn.nci.nih.gov/), was used to identify breast cancer biomarkers currently under investigation.

[1] Shaheed, S. U., Rustogi, N., Scally, A., Wilson, J.*, et al.*, Identification of stage-specific breast markers using quantitative proteomics. *J Proteome Res* 2013, *12*, 5696-5708.

[2] Huang da, W., Sherman, B. T., Lempicki, R. A., Systematic and integrative analysis of large gene lists using DAVID bioinformatics resources. *Nat Protoc* 2009, *4*, 44-57.

[3] Szklarczyk, D., Franceschini, A., Wyder, S., Forslund, K.*, et al.*, STRING v10: protein-protein interaction networks, integrated over the tree of life. *Nucleic Acids Res* 2015, *43*, D447-452.

[4] Krogh, A., Larsson, B., von Heijne, G., Sonnhammer, E. L., Predicting transmembrane protein topology with a hidden Markov model: application to complete genomes. *J Mol Biol* 2001, *305*, 567-580.
