# Supplementary material for: Nipple aspirate fluid—A liquid biopsy for diagnosing breast health
Source: Proteomics Clin Appl. 2017 Jun 26;11(9-10):1700015. doi: 10.1002/prca.201700015 (PMC5638085; doi:10.1002/prca.201700015)
Supplement: Supplementary file 2 — Supplementary Figures 1, A – C. SDS PAGE analysis of matched pairs of NAF samples from additional patients and a healthy volunteer (Cases 5 to 15), N = NAF samples from normal breast, D = sample from diseased breast. Arrow indicates the position of serum albumin Supplementary Figure 2. Correlation of NAF profile pairs, HV = healthy volunteer, PB = patient with benign lesion, PD = patient with DCIS, PI = patient with invasive carcinoma Supplementary Figure 3. Western blot analysis of β‐actin and CYP3A4 presence in the NAF samples. HV = healthy volunteer, PB = patient with benign lesion, PD = patient with DCIS, PI = patient with invasive carcinoma. N = healthy/normal breast, T = tumour [file PRCA-11-na-s002.pptx]

## Slide 1
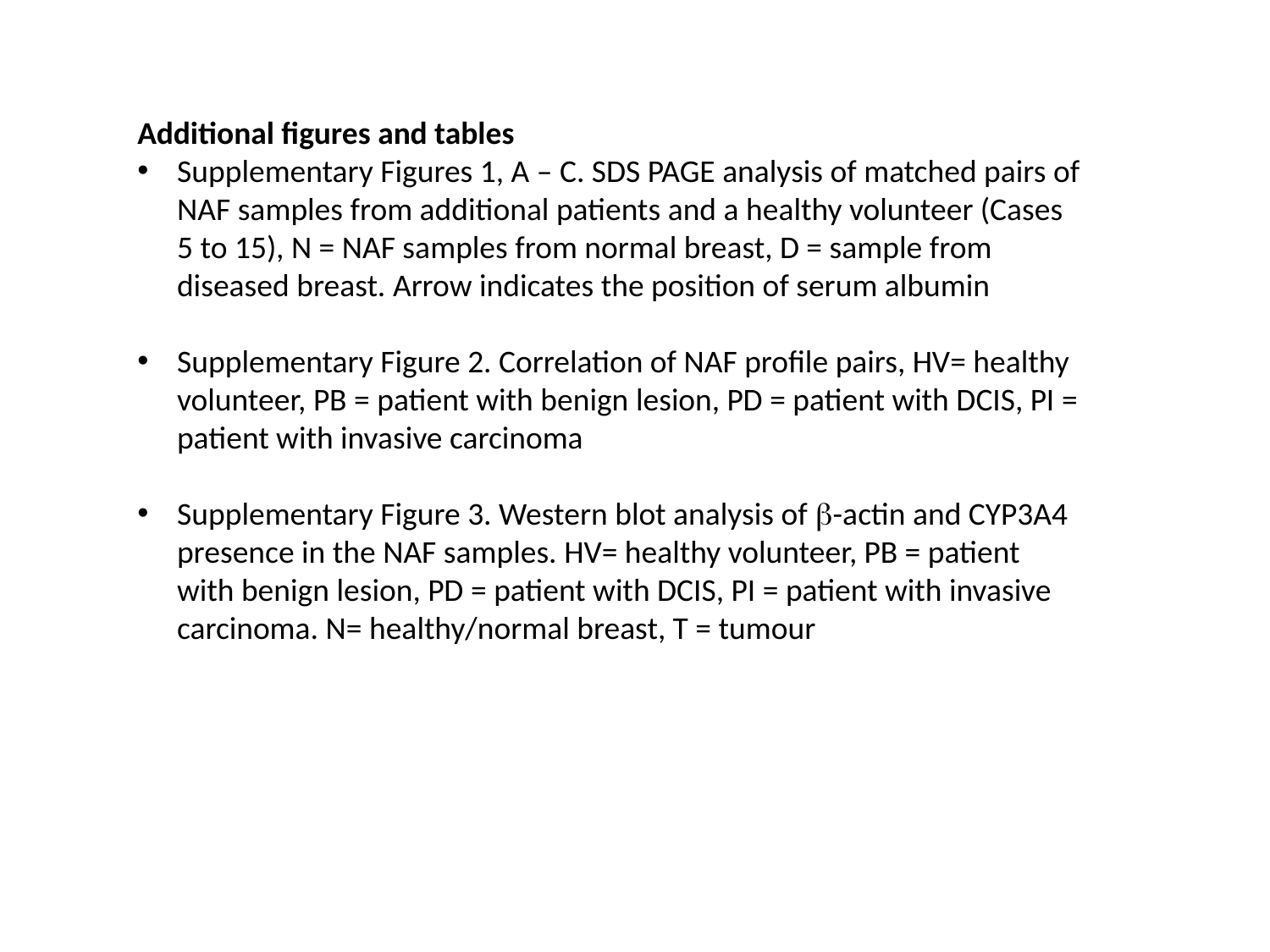

Additional figures and tables
Supplementary Figures 1, A – C. SDS PAGE analysis of matched pairs of NAF samples from additional patients and a healthy volunteer (Cases 5 to 15), N = NAF samples from normal breast, D = sample from diseased breast. Arrow indicates the position of serum albumin
Supplementary Figure 2. Correlation of NAF profile pairs, HV= healthy volunteer, PB = patient with benign lesion, PD = patient with DCIS, PI = patient with invasive carcinoma
Supplementary Figure 3. Western blot analysis of b-actin and CYP3A4 presence in the NAF samples. HV= healthy volunteer, PB = patient with benign lesion, PD = patient with DCIS, PI = patient with invasive carcinoma. N= healthy/normal breast, T = tumour

## Slide 2
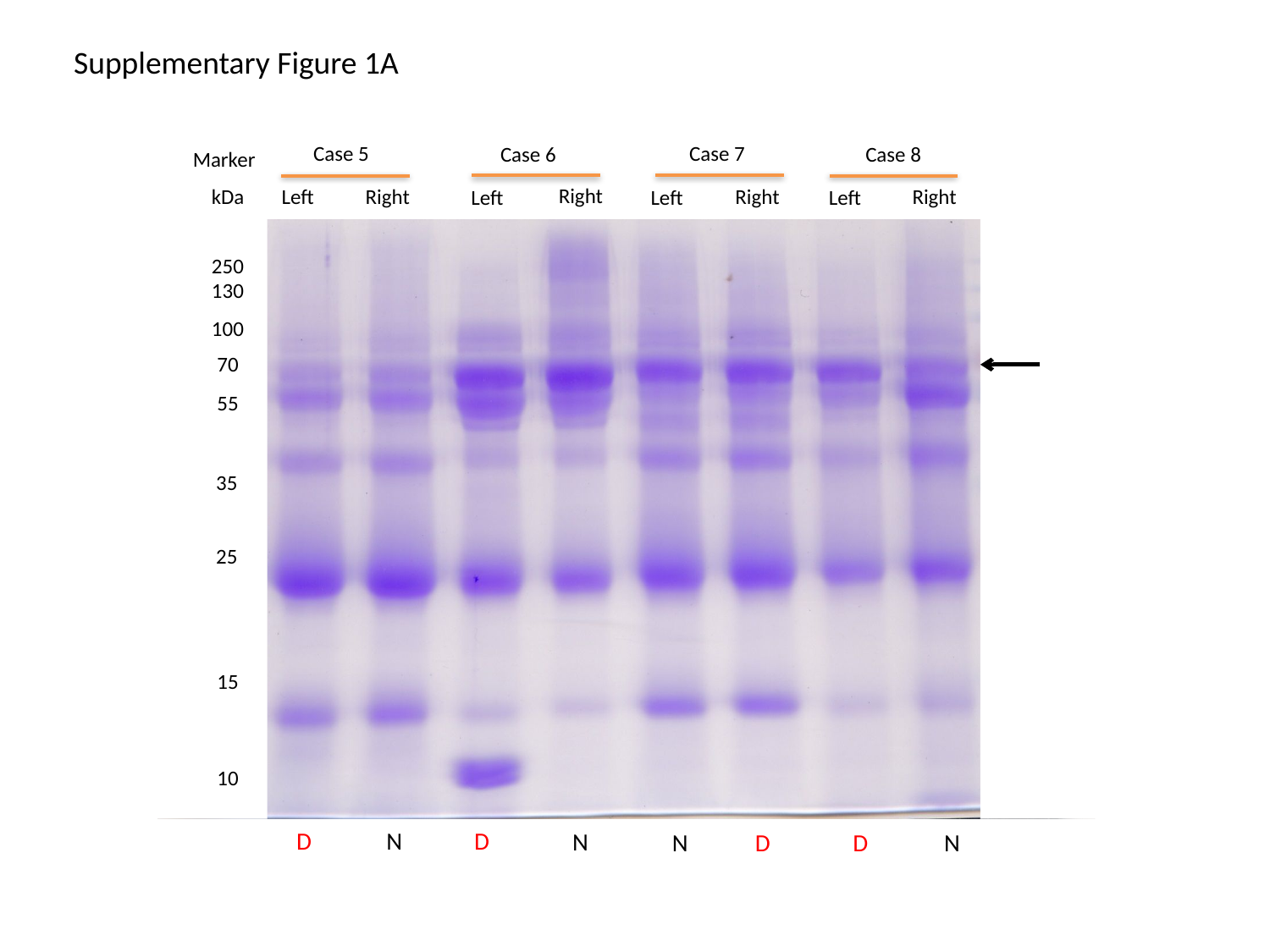

Supplementary Figure 1A
Case 7
Case 5
Case 8
Case 6
Marker
Right
Right
Right
Right
Left
kDa
Left
Left
Left
250
130
100
70
55
35
25
15
10
D
D
N
N
N
D
D
N

## Slide 3
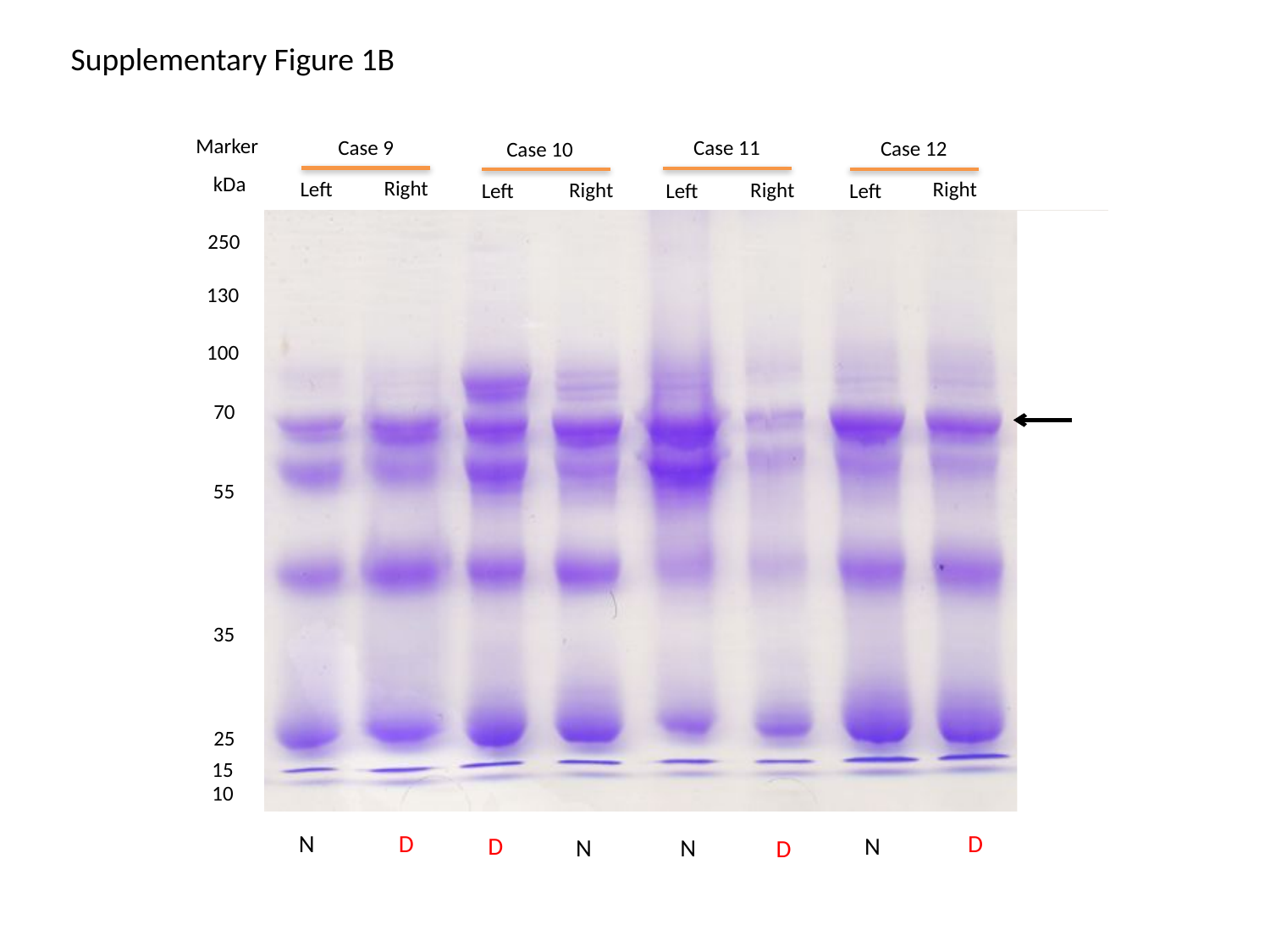

Supplementary Figure 1B
Marker
Case 9
Case 11
Case 12
Case 10
kDa
Right
Left
Right
Right
Right
Left
Left
Left
250
130
100
70
55
35
25
15
10
D
N
D
N
D
N
N
D

## Slide 4
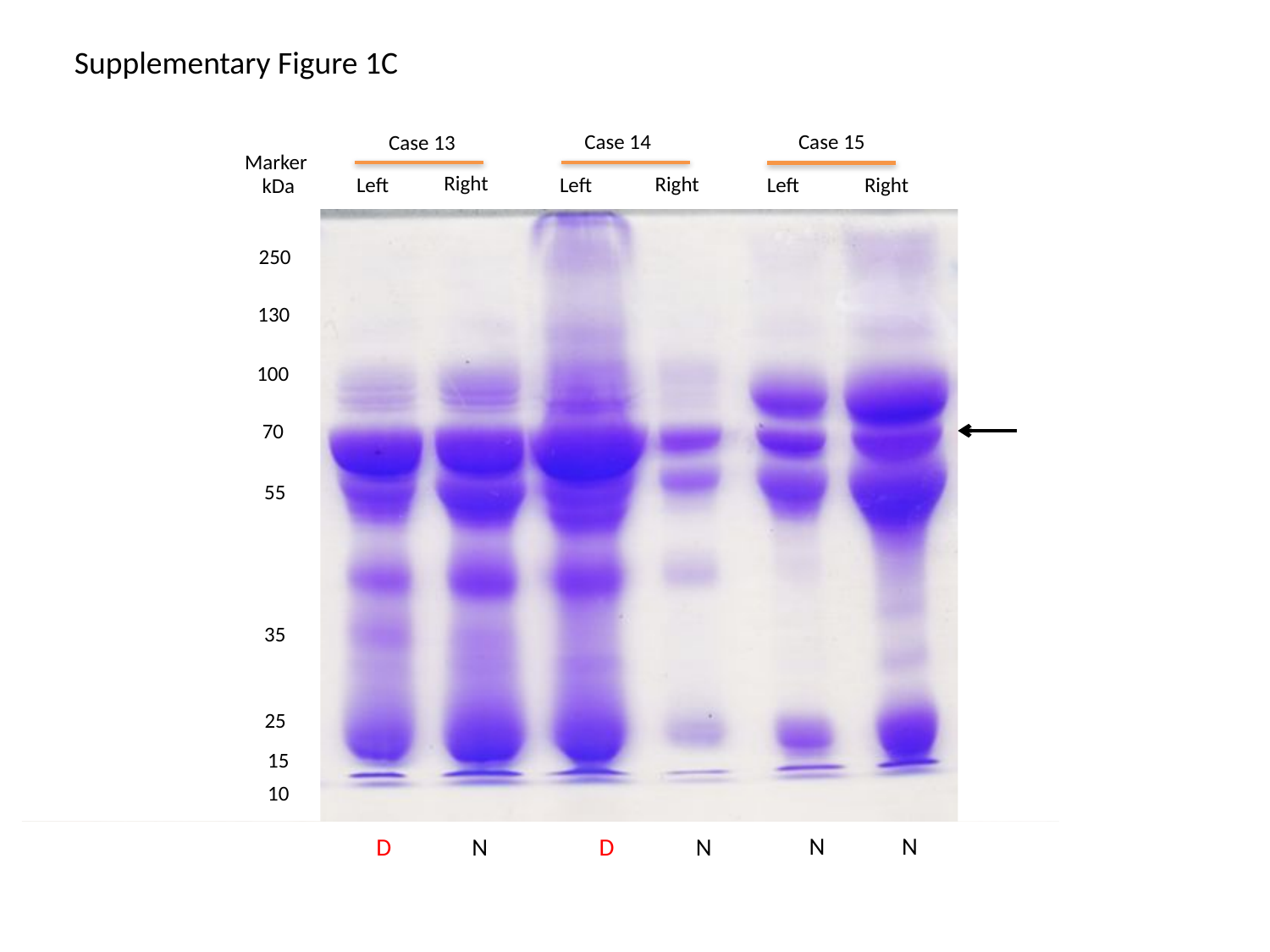

Supplementary Figure 1C
Case 15
Case 14
Case 13
Marker
Right
Right
Left
Right
Left
Left
kDa
250
130
100
70
55
35
25
15
10
N
N
D
N
D
N

## Slide 5
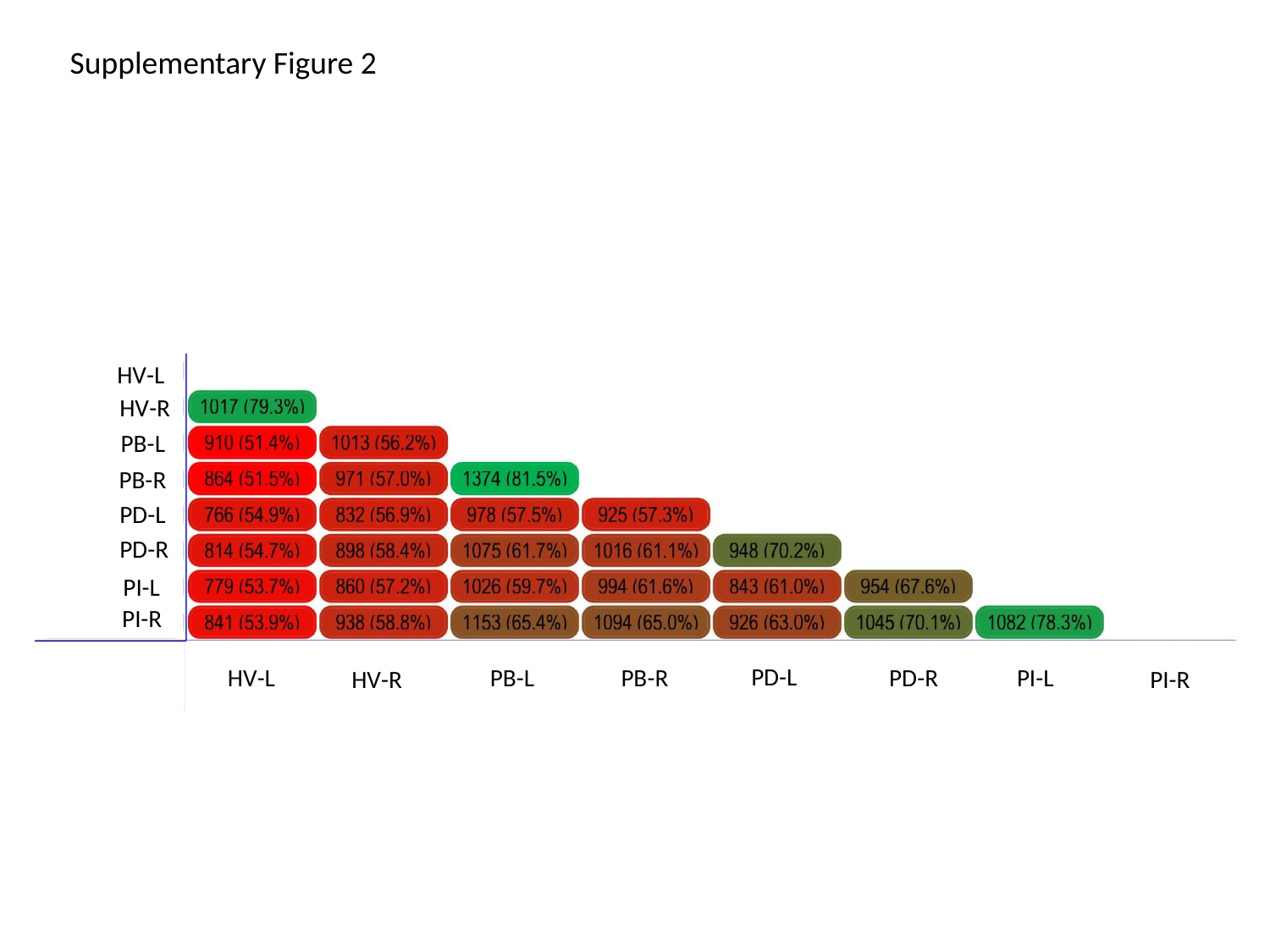

Supplementary Figure 2

## Slide 6
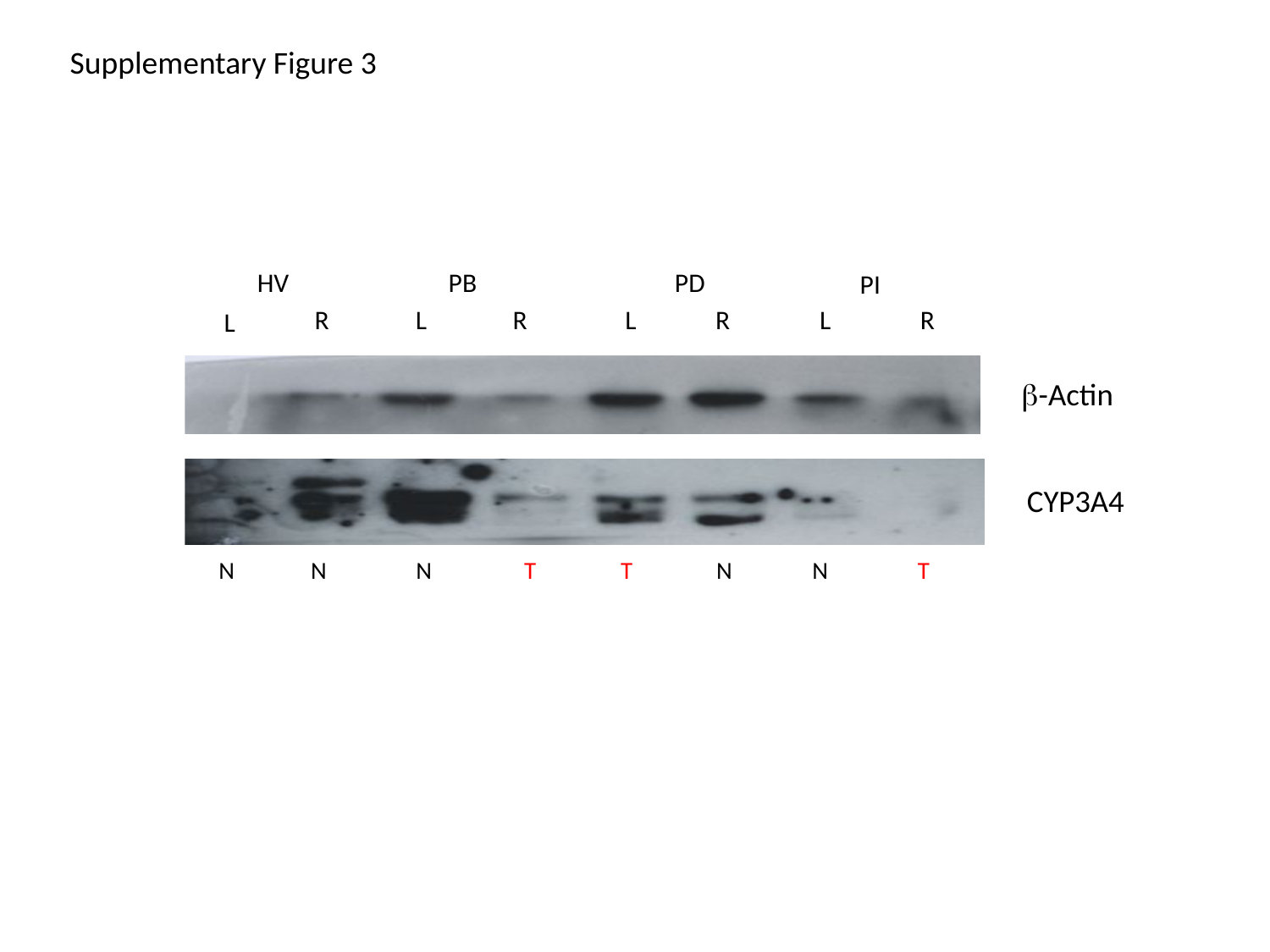

Supplementary Figure 3
